# Imaging-guided radiofrequency ablation of osteoid osteoma in typical and atypical sites: Long term follow up

**DOI:** 10.1371/journal.pone.0248589

**Published:** 2021-03-18

**Authors:** Francesco Somma, Vincenzo Stoia, Roberto D’Angelo, Francesco Fiore

**Affiliations:** Radiologia Interventistica, Istituto Nazionale Tumori IRCCS “Fondazione G. Pascale”, Napoli (IT), Naples, Italy; Kanazawa University, JAPAN

## Abstract

**Purpose:**

To assess efficacy and safety of imaging-guided radiofrequency ablation (RFA) of Osteoid Osteoma (OO) in both typical and atypical sites.

**Methods and materials:**

Between January 2014 and March 2019, 102 consecutive percutaneous RFA were performed and retrospectively reviewed. The procedures were performed using a RFA bipolar ablation system (Covidien, exposed tip of 0.7-1cm), under Computed Tomography (CT) guidance or using a navigation system (Masmec) under CT and Cone Beam CT (CBCT) guidance. Patients were followed up over 24 months. Clinical success and recurrences were considered on the base of established criteria. In patients with clinical failure and/or imaging evidence of relapse, retreatment was considered.

**Results:**

Administered power per-procedure was ≤8 W (mean temperature, 90°C). The pre-procedure average value of visual analog scale (VAS) was 8.33+/-0.91. Primary and secondary success rate 96.08% (98/102) and100% (102/102), respectively. No major complication was described. Technical success was proved in every patient by CT scan acquisition after needle positioning. Relapse and tumour location were significantly correlated (p-value = 0.0165). The mean dose-length product was 751.55 mGycm2. Advanced bone healing was noted in 68 lesions after 1y-follow up and in 86 lesions after 2y-follow up.

**Conclusion:**

Imaging-guided percutaneous RFA is a highly effective technique for OO, both in typical and atypical sites. CT or CBCT guidance, navigation systems and operator experience grant the technical success, which is the most crucial parameter affecting outcome.

## Introduction

Osteoid osteoma (OO) is a benign bone tumor (10% of benign bone tumors), composed of a central nidus generally of less than 15 mm in diameter and surrounded by osteoblasts and peripheral reactive zone of thickened cortical or trabecular bone and loose fibro vascular tissue [1]. It is more frequent in males, usually in young adults [2].

The most common symptom is bone pain with characteristic nocturnal exacerbation, usually relieved by salicylates or non-steroidal anti-inflammatory drugs administration. Painful OOs may be found in the epiphysis, metaphysis, or diaphysis, and may involve the cortex or cancellous bone [3]. The appendicular skeleton is the most frequent location, in particular femur and tibia. However, rare location is also possible and is known as atypical location. Growth disturbance, scoliosis, osteoarthritis, located within the capsule of a joint, swelling, synovitis, restricted movement, and contracture are rare manifestations [4].

The classic radiographic appearance is of a small central radiolucent nidus surrounded by a zone of bony sclerosis and cortical thickening caused by endosteal and subperiosteal new bone formation [4]. Computed Tomography (CT)is the method of choice to confirm the diagnosis and to show the cortex integrity and exact location and size of the nidus. Magnetic Resonance (MR) imaging is quite sensitive but non-specific. MR better shows osteoid osteomas located in cancellous bones and the associated soft tissue and intramedullary changes [5]. Tc 99-HDP bone scintigraphy may also be performed, showing intense radiotracer uptake in the region of nidus and surrounding bone with a typical double density sign [6].

The standard treatment traditionally had been surgical excision [1]. However, along with long-term analgesia it had important disadvantages: difficulties to identify the nidus during surgery and to reach the lesion in deep locations such as hip joint, higher aggressiveness, higher risk of post-operative complications including stress fractures and a prolonged hospital stay [7].

Ever since Rosenthal et al. [8] reported the efficacy of CT-guided thermal ablation in OO, there has been a steady paradigm shift towards minimally invasive percutaneous treatment options such as interstitial laser ablation [9], cryotherapy [10–12] and especially radiofrequency ablation (RFA) [13–15].

Herein, we present our oncologic center experience to assess efficacy and safety of CT guided RFA in the treatment of OO located both in typical and atypical sites, trying to answer the question whether or not OO in typical and atypical sites have different prognosis.

## Material and methods

### Ethics statements

This study has been approved by the Scientific Committee of our National Cancer Institute. Appropriate written informed consent was collected before every procedure. All data were collected retrospectively by a dedicated data managerin an approved protocol. All authors reviewed and approved this manuscript.

### Patients

**Fig 1:**
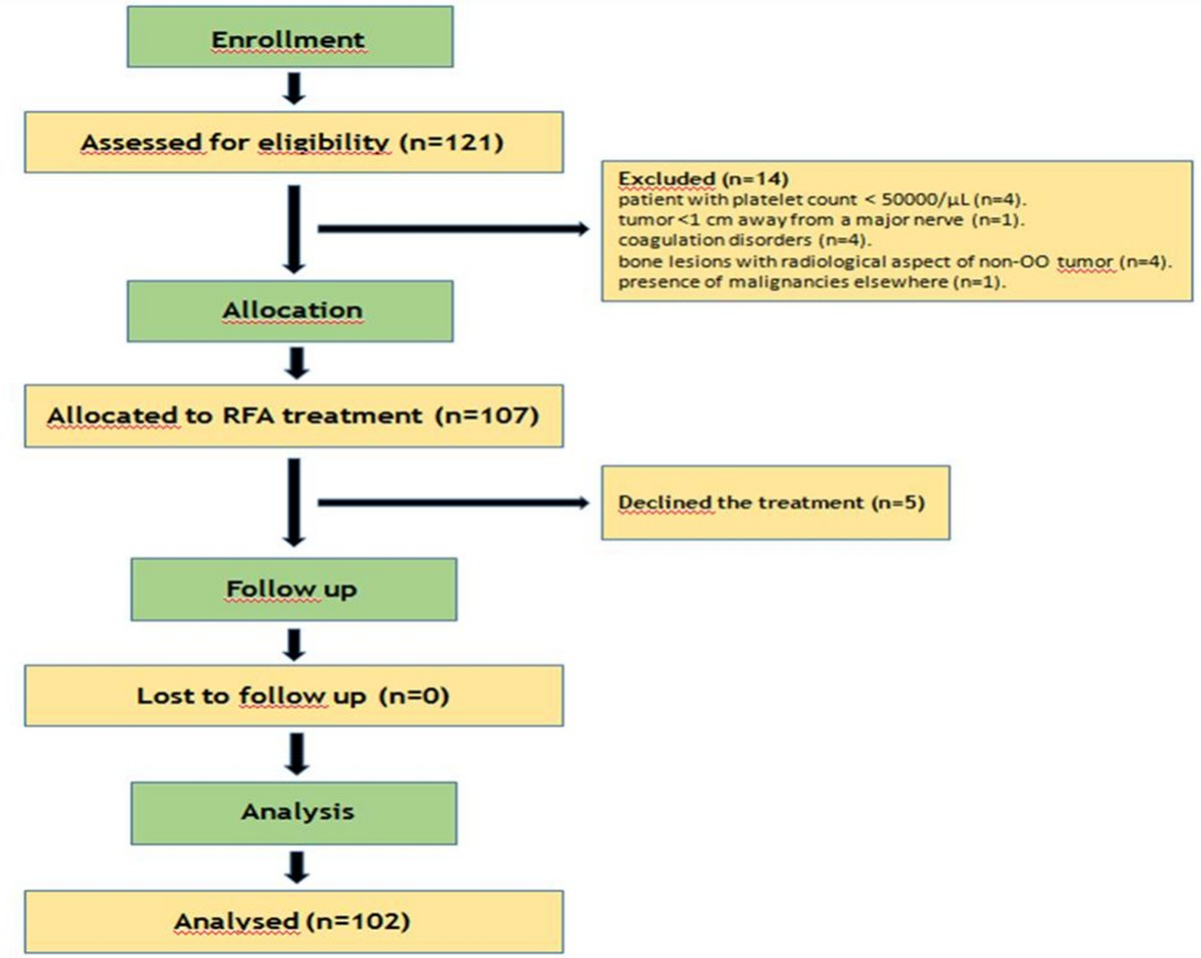
Consort flow chart.

Between January 2014 and March 2019, 121 consecutive patients were evaluated in our Interventional Radiology Department for chronic bone pain with nocturnal exacerbation relieved by salicylates. Inclusion criterion was the presence of a bone lesion with both clinical and radiological aspect of OO. Nineteen (15.70%) patients were excluded (14 for not meeting inclusion or meeting exclusion criteria; 5 declined the treatment). Exclusion criteria were: patient with platelet count < 50000/μL(4); tumor <1 cm away from a major nerve (1) due to probable neural damage; coagulation disorders (4); bone lesions with radiological aspect of non-OO tumour (4); presence of malignancies elsewhere (1). One patient with other malignancy and one with low platelet count had been previously operated with Trans-Arterial Ethanol-Lipiodol Embolization (TAELE) [16] and Trans-Arterial Radio-Embolization (TARE) [17], respectively. The remaining 102 patients (72.55% male; 27.45% female; range of age 14-71years) were diagnosed with OO on the base of clinical examination and imaging. All patients underwent bone biopsy within the treatment. Written informed consent was obtained before each procedure. All 102 patients included in the series had a histological diagnosis of OO based on biopsy. The flow chart of our study is shown in *Fig 1*.

### Imaging

Medical records and imaging were reviewed for size of the radiolucent nidus, location, and whether the lesion was intra-cortical or trabecular and intra- or extra-articular. All patients underwent plain film radiographs before ablation. Other imaging modalities for diagnosis were multi-detector CT (pediatric = 4/12, 33.33%; adult = 75/95, 78.95%), MRI (pediatric = 9/12, 75.0%; adult = 30/95, 31.58%) and Tc 99-HDP bone scintigraphy (pediatric = 0.0%; adult = 12/95, 12.63%). Follow up imaging was reviewed to establish whether the ablation was complete. If a patient underwent reintervention, the new imaging was reviewed for evidence of new or recurrent lesion.

### Pain

Visual Analog Scale (VAS) for numeric pain score (0–10) was recorded at presentation andduring follow up. Response to analgesics was also recorded.

### Technique

**Fig 2:**
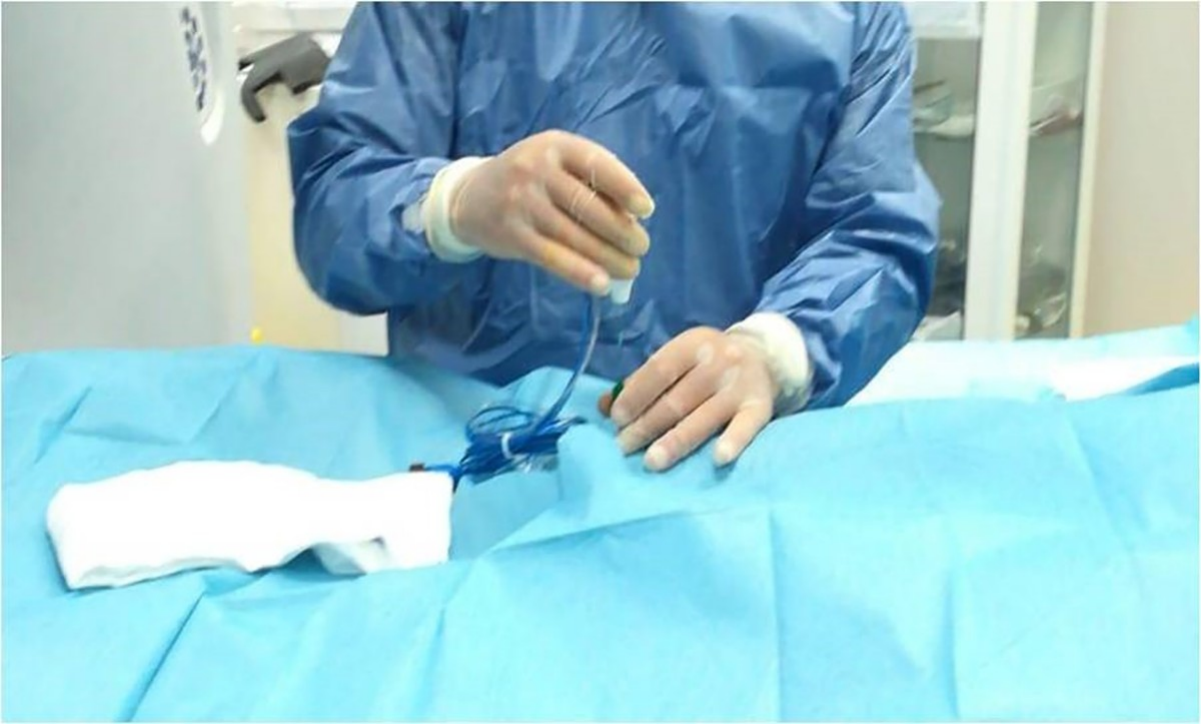
The RF electrode positioning in the osteoid osteoma before ablation under CT guidance.

Two operators with similar experience levels treated all patients after collection of informed consent. Spinal or epidural anesthesia was performed in most patients. No patient was treated under general anesthesia. Peripheral nerve anesthesia was conducted under ultrasound guidance in patients with OO in the extremities. Local anesthesia (2–5 ml of mepivacaine hydrochloride at 2%) was performed in the cutaneous site of needle penetration. Following, unenhanced CT was performed to localize the lesion and plan the approach to avoid vital structures. *Fig 2* shows the RF electrode positioning in the osteoid osteoma before ablation under CT guidance. In case of OO placed in hard-to-access locations, a navigation system (Masmec manufactured) was used under Cone Beam CT (CBCT) guidance in order to spare time of intervention and dose administered to patients. After choosing the insertion point on multiplanar reconstructions (MPR), the position was ascertained by CT using a radiopaque landmark and the skin was marked. A stab incision was made and a biopsy trocar (Bone Biopsy System, Bonopty, Radi Medical Device, Sweden) was advanced to cortex. Drilling through the cortex was mandatory in cases of dense cortical bone (Bonopty drill set, standard length 122 mm, extended length 160 mm, calibre 17 G12/1.7-mm). After reaching the nidus, in all patients tissue sample was obtained for histology using a biopsy needle (Bonopty biopsy needle, Radi Medical Device, Sweden) inserted through the trocar. Next, the electrode was inserted through the trocar aiming at the center of the nidus of its active tip under imaging-guidance. A RF bipolar ablation system (Covidien) was used to perform RFA by raising the temperature directly to 90–93°C for around 6 min.

### Follow up

As per the protocol, the baseline (T0) was considered as the day before the treatment. Afterwards, patients were followed up in the outpatient department (OPD) 1 month (T1),3 months (T2) and 12 months (T3) after the procedure. The VAS pain score was obtained by interview, and the change in analgesic intake following the procedure was assessed. Patients with recurrent/residual symptoms were identified and considered for RFA retreatment.

### Preprocedure

Detailed history was obtained from the patients regarding the duration of illness, treatment underwent, etc. Severity of pain was assessed subjectively using the VAS score. Preprocedural workup included a coagulation profile including prothrombin time/International normalized ratio (INR) and pre-anesthetic workup including blood investigations, chest radiograph, and clinical exam by the anesthetist. Plain radiographs and non-contrast CT of the bone lesion were performed in all cases to establish the diagnosis. In inconclusive cases, bone scintigraphy or MRI was performed additionally.

### Postprocedure care

The patients were observed in the procedure room until there was complete recovery from anesthesia and establishment of spontaneous breathing. Oral or intramuscular analgesics were administered because patients often had increased need of analgesia in the immediate peri-operative period. The patients were discharged the day after the procedure. Patients with lesions in the weight bearing bones were instructed to restrain from strenuous activities for a minimum of 1 month. Otherwise, patients were not restricted from normal daily activities.

### Definitions

Technical success was defined if the electrode was placed so that no portion of the lesion was more than 5–7 mm away from the exposed tip and if the target energy was deposited [3]. Residual symptoms were defined as pain or impaired function or both identical to the presenting complaints that persisted for more than 2 weeks after radiofrequency thermal ablation. Recurrent symptoms were defined as the reappearance of symptoms that followed a symptom-free period after RF thermal ablation [15].

### Clinical outcome

Technical success, reintervention rate, primary and secondary clinical successes, and total follow-up time were recorded. Primary clinical success was defined as VAS of 1.5/10 or less after a single ablation. Secondary clinical success was defined as visual analog scale of 1.5/10 or less following repeated ablations.

The patients were categorized into three groups, as described by Rosenthal et al. [3]: clinical success–pain free and did not require medications/additional procedures; indeterminate–pain was neither severe enough nor frequent enough to necessitate additional investigations or procedures; clinical failure–recurrent/persistent pain requiring analgesic intake and additional procedures.

### Statistical analysis

The statistical analyses were performed using Matlab statistical toolbox version 2008 (MathWorks, Natick, MA, USA) for Windows at 32 bit, on a random sample of 102 patients, comparing different sub-groups with different characteristics. ANOVA test for repeated measures was used in multicomparison among means, when the same parameter had been measured in different conditions on the same subjects, and p-value was computed with Bonferroni corrected for pairwise comparisons. Different time points were fixed to manage patients’ follow up: T0 –baseline time, immediately before the procedure; T1–1-month follow up; T2–3-month follow up; T3–12-month follow up. Univariate and multivariate linear correlation analyses were performed using the t-Student test for analyzing respectively the Pearson’s linear correlation coefficient (R) and the partial correlation coefficient (Rp), under null hypothesis of Pearson’s linear correlation coefficient R = 0.

All statistical tests with p-value < 0.05 were considered as significant.

## Results

The characteristics of the patients are given in *Table 1*. Of 102, there were 12 (11.76%) pediatric patients (≤16y), 30 (29.41%) young adults (>16y; ≤21y) and 60 (58.82%) adults (>21y). Among pediatric patients, there were 7 (6.86%) male and 5 (4.90%) female with a mean age of 15.08±0.99 (range 13–16). Among adult patients (>16y), there were 62 (60.78%) male and 28 (27.45%) female patients with a mean age of 32.09±15.20 (range 17–75). Mostly, patients presented with chronic pain at the lesion site, and a large part of both pediatric (8/11; 72.72%) and adult (75/92; 81.52%) patients complained of night pain. Oral analgesics (Aspirin, Ibuprofen, Naproxen and others) were effective in 9/11 (81.81%) of pediatric and 71/92 (77.17%) of adult patients. The mean total ablation time was 7.2 ± 3.4 min (range, 3–15 min). No major complication was registered. Transient and bearable skin burns and muscle local pain were referred by patients in our series in 5/102 (4.90%) and 1/102 (0.98%), respectively. The mean dose-length product per-procedure was 751.55 mGycm2.

**Table 1:** Demographics data of the patients.

Parameters	Value
***Age*** (y)	
Age (years), mean ± SD (range)	30.09 ± 15.23
≤ 21years	42/102 (41.18)
>21 years (adult)	60/102 (58.82)
≤ 16 years (paediatric)	12/102 (11.76)
> 16 years	90/102 (88.24)
> 16 years; ≤ 21years	30/102 (29.41)
***Sex***	
Male	74/102 (72.55)
Female	28/102 (27.45)
***Imaging for Diagnosis***	
CT	79/102 (77.45)
MRI	39/102 (13.73)
MRI + CT	8/102 (7.84)
Tc 99-HDP Bone Scintigraphy	12/102 (11.76)
***Nidus diameter*** (cm), mean ± SD (range)	0.58 ± 0.27
***Medicaments***	
Aspirin	61/102 (59.80)
Ibuprofen	21/102 (20.59)
Naproxen	15/102 (14.70)
Others	5/102 (4.90)
***Pain duration before treatment*** (months), mean ± SD (range)	7.21± 2.24

SD, standard deviation.

CT, Computed Tomography.

MRI, Magnetic Resonance Imaging.

The anatomical distribution of the lesions in the body was described in *Table 2:* lower extremities (72/102, 70.59%) and trunk skeleton (21/102,20.59%) were the most frequent locations. Lesions were atypically located in 25 patients (25/102, 24.51%). As shown in *Table 3A*, technical success was of 100%, with a primary and secondary clinical success rate of 96.08% and 100%, respectively. Among patients in our study, only 4 newly experienced pain after CT-guided RFA, which means an overall rate of relapse of 3.92%. However, after population stratification for typical and atypical lesion location, clinical relapse rate was 0% for lesions in typical sites and 11% (4 patients) for lesions in atypical sites. Among these, in one case the pain was not due to recurrence but to a new lesion.

**Table 2:** Location of osteoid ostema in the study population.

Anatomical location of Osteoid Osteoma		
**Location of the Lesion**	**Number (102)**	**%**
*Talus	1	0.98
* Rib	1	0.98
* Sacrum	1	0.98
* Iliac crest	1	0.98
* Sacroiliac	1	0.98
* Pedicle of L1	1	0.98
* Pedicle of L4	1	0.98
* Olecranon	1	0.98
Fibula	1	0.98
Radius	1	0.98
* Acetabulum	2	1.96
* Transverse process of L5	2	1.96
* Iliac spine	2	1.96
* Ischium	3	2.94
Humerus	3	2.94
* Iliac wing	6	5.88
Tibia	26	25.49
Femur	46	45.10
**Side of the Lesion**	**Number (102)**	**%**
Left	50	49.02
Right	52	51.98
**Site of the Lesion**	**Number (102)**	**%**
Typical	77	75.49
Atypical	25	24.51

L, lumbar vertebra.

*, Atypical locations.

**Table 3:** a. Results after Treatment. b. Trend of VAS.

**Parameters**	**Value**
***VAS score***, mean ± SD (range)	
Pre-procedure (T0)	7.09 ± 0.33
1-month follow up (T1)	1.49 ± 0.83
3-month follow up (T2)	0.61 ± 0.49
12-month follow up (T3)	0.47 ± 0.45
***Adverse events***, n (%)	
Transient skin burn	5/102 (4.90)
Transient local muscle pain	1/102 (0.98)
***Relapse***, n (%)	
Typical site OO	0/102 (00.00)
Atypical site OO	3/102 (2.94)
New lesion	1/102 (0.98)
***Final Result***, n (%)	
Technical success	102/102 (100)
Primary Clinical success	98/102 (96.08)
Secondary Clinical success	102/102 (100)
***Pain duration before treatment*** (months), mean ± SD (range)	7.21± 2.24
**Trend of VAS (statistical analysis)**	
**Hypothesis**	**p-value**
μ (T0)>μ (T1)	<0.001* (A)
μ (T0)>μ (T1)	<0.0001* (B)
μ (T0)>μ (T2)	<0.0001* (B)
μ (T0)>μ (T3)	<0.0001* (B)
μ (T1)>μ (T2)	<0.0001* (B)
μ (T1)>μ (T3)	<0.0001* (B)
μ (T2)>μ (T3)	1.0 (B)

SD, standard deviation.

* significant test.

A, ANOVA test for repeated measures.

B, Bonferroni corrected p-value.

T0, baseline.

T1, one month after treatment.

T2, three months after treatment.

T3, twelve months after treatment.

**Fig 3:**
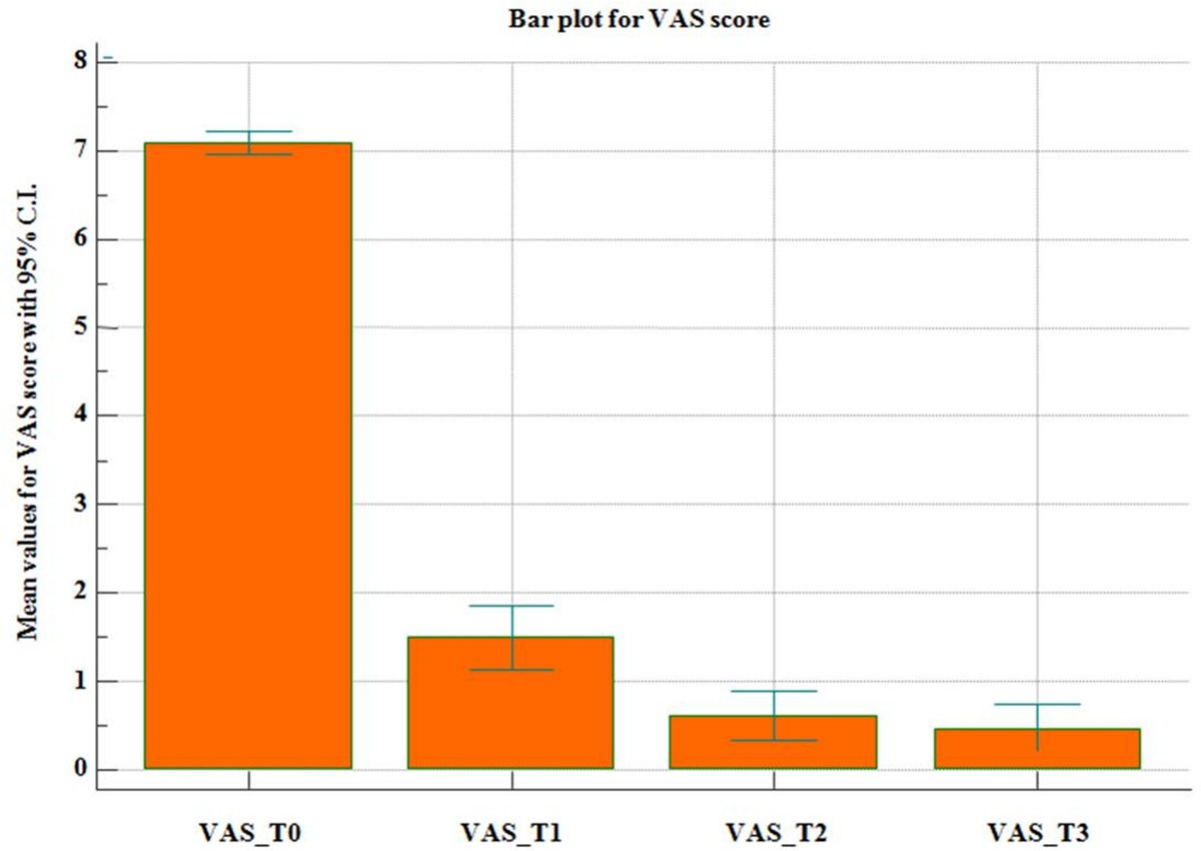
Average values for VAS score with 95% C.I.

The VAS score was used to evaluate pain intensity before and after the treatment. Its trend during the follow up period is shown in *Fig 3*. In addition, *Table 3A* and *3B* illustrate the statistical analysis performed on the VAS change after the treatment. In particular, a statistical significant difference was observed between T0and T1(baseline vs. one month after treatment: 7.09 > 1.49, p-value <0.0001) and between T1 and T2 (one month after treatment vs. three months after treatment: 1.49>0.61, p-value <0.0001). Instead, no statistical significant difference was evident between T2 and T3 (three months after treatment vs. twelve months after treatment: 0.61>0.47). *Table 3A* also reports the adverse events and relapse rates.

A linear correlation analysis between dependent and independent variables was performed and displayed in *Table 4*. The whole pain perceived by patients according to the VAS scoring, before and after the treatment, was found to be significantly correlated to various factors: a) age, (R = 0.385; p-value <0.0001), elderly patients suffered more pain than young patients; b) nidus size (R = 0.225; p-value = 0.0229), the bigger was the lesion the higher VAS was registered by patients; c) lesion location (R = -0.228; p-value = 0.0213), a significant negative correlation was found between suffered pain and lesion location, thus meaning that atypically located OO resulted to be more painful. The multivariate analysis confirmed that the overall pain complained by patients was significantly correlated to age of the patient (Rp = 0.321; p-value = 0.0016) and location of the lesion (Rp = -0.218; p-value = 0.00345).

**Table 4:** Linear correlation analysis between dependent and independent variables.

Linear correlation analysis	Univariate analysis	Multivariate analysis
	**R (p-value)**	**Multiple linear correlation coefficient = 0.482**
*Total VAS/Age*	0.385 (0.0001) *	Rp = 0.321; p-value = 0.0016 *
*Total VAS/Gender*	0.048 (0.631)	Rp = 0.026; p-value = 0.0806
*Total VAS/Location*	-0.228 (0.0213) *	Rp = -0.218; p-value = 0.00345 *
*Total VAS/Size at T0*	0.225 (0.0229) *	Rp = 0.115; p-value = 0.270
*Total VAS/Relapse*	-0.077 (0.440)	Rp = 0.072; p-value = 0.492
	**R (p-value)**	**Multiple linear correlation coefficient = 0.997**
*Relapse/Age*	0.004 (0.968)	Rp = 0.005; p-value = 0.962
*Relapse/Gender*	-0.036 (0.722)	Rp = -0.132; p-value = 0.204
*Relapse/Location*	-0.237 (0.0165) *	Rp = -0.080; p-value = 0.446
*Relapse/Size at T0*	0.106 (0.290)	Rp = -0.285; p-value = 0.0054 *
*Relapse/Durationof the treatment*	0.049 (0.627)	Rp = -0.139; p-value = 0.182
*Relapse/Administrated Power*	-0.013 (0.899)	Rp = 0165; p-value = 0.112
*Relapse/Retreatment (RFA)*	0.997 (<0.0001) *	Rp = 0.997; p-value < 0.0001 *
*Relapse/Total VAS*	-0.077 (0.440)	Rp = 0.072; p-value = 0.492

* significant test.

R, Pearson’s linear correlation coefficient.

Rp, partial correlation coefficient.

T0, baseline.

RFA, Radiofrequency Ablation.

Furthermore, atypically located OO were found to be more likely associated to relapse (R = -0.237; p-value = 0.0165) and retreatment (R = -0.233; p-value = 0.0185): tumours located in less frequent sites such as talus or rib entails a higher rate of relapse, if compared to more frequent location such as the femoral bone.

On the contrary, no significant statistical association has been proved between suffered pain and sex (R = 0.048; p-value = 0.631), nor between baseline pain and relapse (R = -0.077; p-value = 0.440). Also, no correlation has been found between relapse and the following parameters: age (R = 0.004; p-value = 0.968), sex (R = -0.036; p-value = 0.722), baseline size of the nidus (R = 0.106; p-value = 0.290), duration of the treatment (R = 0.049; p-value = 0.627), administrated power (R = -0.013; p-value = 0.899).

With regard to bone healing, advanced bone healing was noted in 68 lesions after 1y-follow up and in 86 lesions after 2y-follow up. Minimal and absent bone healing after 2y-follow up was found respectively in 13 and 3.

## Discussion

This study presents a single-center large series of CT-guided RFA of OO, both in typical and atypical sites. All cases were analyzed with regard to localization, volume of the lesion, symptoms, RFA technique, therapeutic success and clinical follow up.

Our technical and clinical success were comparable to those assessed in previous published studies, ranging from 91–95% [3,12,18,19]. In particular, Kulkarni et al. [18] recorded a technical success rate of 100%, with a primary and secondary clinical success rate of 97.7%, and 100%, respectively. Rehnitz et al. [19] reported primary and secondary success rates of 96.1% and 100%, respectively.

The location of typical and atypical OO lesions resulted to be similar to the described distribution frequency in the different body regions [20,21].

Differently from other studies describing different pain characteristics for typical and atypical OO [22,23], our patients reported a similar depiction of their pain, both for typically and atypically located lesions. The pain was always described as clearly localized. Only a slight percentage of atypically located OO showed pain radiating to adjacent body regions, mostly in case of lesions in the trunk skeleton, especially spinal column. Indeed, it is well know that atypically located OO could imitate functional symptoms in the area of the supporting structures, even mimicking radicular syndromes [23]. This could explain why many patients reported a long course of disease at their first survey, with up to 8-month period of continuing pain before having a correct diagnosis. Similar observations have already been proposed [18,24]. Our results clearly correlate the pain suffered by patients affected by OO with age, lesion size at baseline and lesion location (atypical site > typical site): elderly patients and patients with bigger and/or atypically located OO complained of stronger pain. On the contrary, the registered pain seemed to be not influenced by gender. With regard to the baseline pain, we also analysed whether there was or not a correlation between the VAS registered at the diagnosis (before the treatment) and the relapse: in our experience, these parameters were not statistically associated, thus meaning that the baseline pain does not represent a useful prognostic factor for relapse.

With regard to the clinical success, seen as the restoration of the patient’s quality of life, or in other words the patient satisfaction, our rate was slightly higher than the average calculated in a metanalysis compiled by Gebauer et al [25]. Indeed, among the 1350 patients considered by Gebauer et al, the success rate was between 65% and 100% (average: 92%), compared to primary and secondary clinical success of 96.08% and 100% in our series. Our result about pain modification is also illustrated in *Fig* 3, showing that in our study population the most significant reduction of pain was observed within the first period of follow up. This is quite common in previous published papers about this topic [18,19,24,25], which describe a drop of pain intensity in the first two weeks after RFA treatment.

Among our patients, only 4 newly experienced pain after CT-guided RFA. All of them had been treated for atypically located OO. Three recurrences and one new lesion were retreated with CT-guided RFA without complications nor further recurrence. At our knowledge, no previous researcher described the case of clinical relapse due to a new lesion diagnosed in a different location few weeks after the first RFA treatment. On the contrary, several previous studies already described recurrences for atypically located OO [3,12,19,26], rating up to 35% of all cases in the series collected in 2013 [25]. Compared to this study, our rate of 11% of atypically located OO (3.92% of all cases) resulted to be definitely lower.

**Fig 4:**
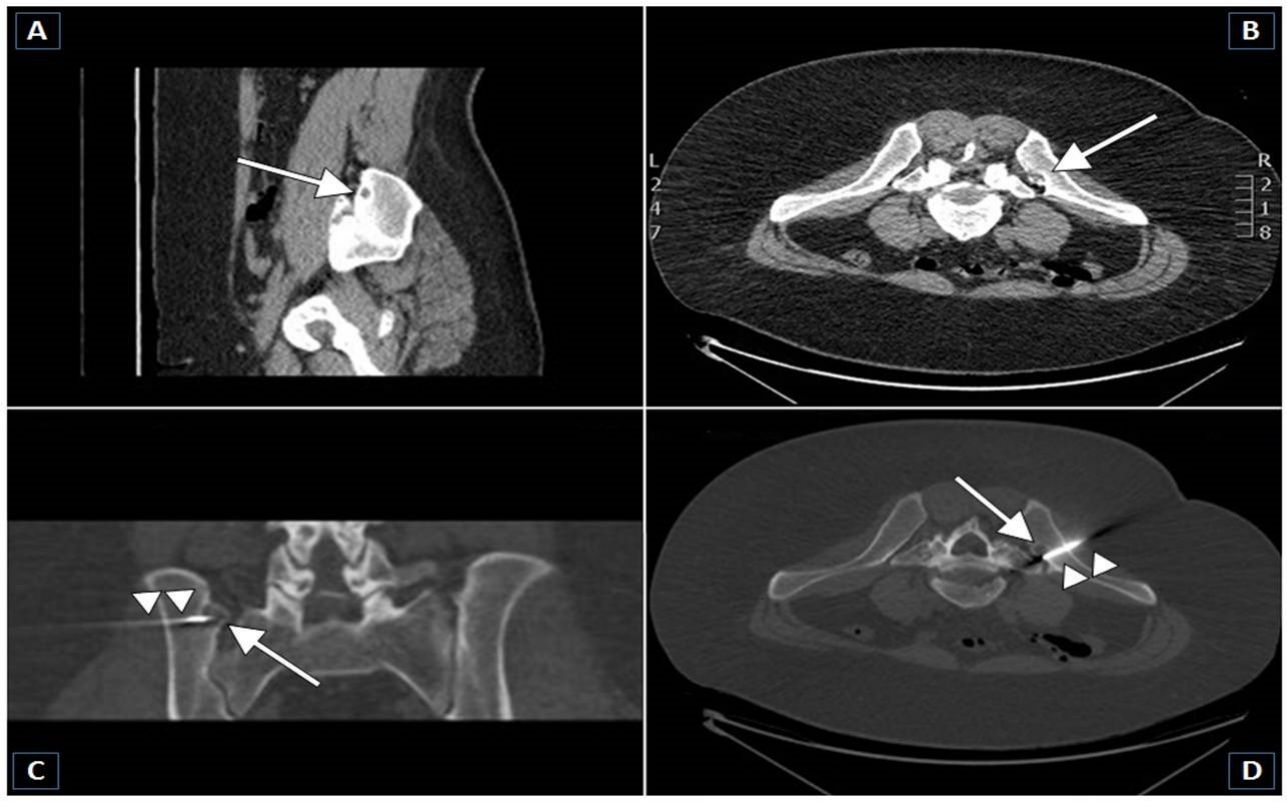
23-year-old male patient with an osteoid osteoma in the left sacroiliac joint. **A, B**–Sagittal and axial CT scans performed with patient in prone decubitus position show an osteoidosteoma in the left sacroiliac joint (arrows). The diameter of the nidus is 13 mm. **C, D**–Coronal and axial CT-guided RFA scans show the correct position of the tip of the RF electrode (arrow heads) within the center of the nidus (technical success). Ablation was successfully performed. No complication was registered. The patient was discharged symptom-free three days after the procedure (clinical success).

**Fig 5:**
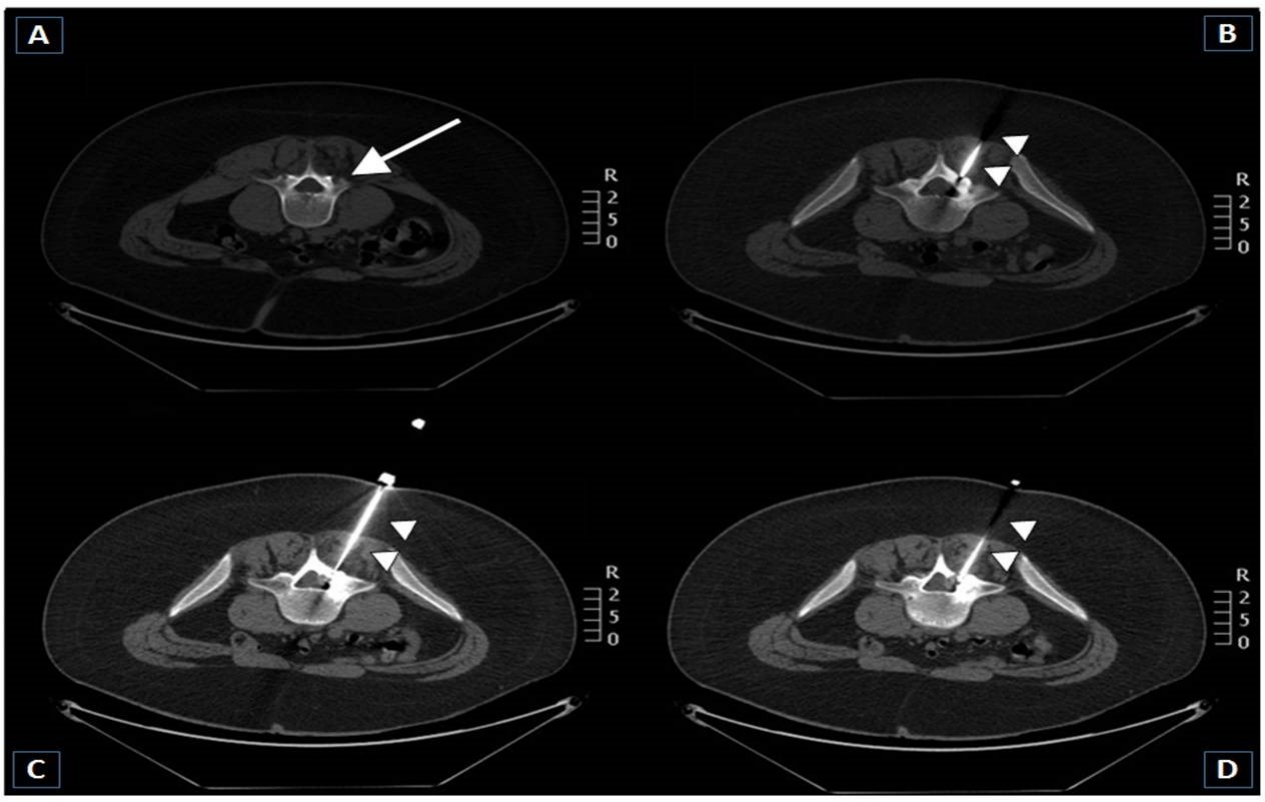
15-year-old male patient affected by an osteoid osteoma in the left lamina of the posterior arch of L4. **A**–Axial CT image shows an osteoidosteoma in the external aspect of the posterior arch of L4. The diameter of nidus is 7 mm. **B, C, D**–Axial CT-guided RFA scans show the correct position of the tip of the RF electrode within the center of the nidus (technical success). (c) Ablation was successfully performed. The risk was the possibility of affection of the nerves belonging to the caudaequina and the left L5 nerve root by thermal injury. However, this did not happen as the size of the active electrodetip was exactly equal to the diameter of the nidus and the whole thermal energy was contained within the nidus. The patient was discharged symptom-free two days after the procedure.

Indeed, many studies have investigated the technical success of the treatment of technically challenging located OOs [18,24,27–32]. Our study shows that image-guided thermal ablation is also well suited for atypically located OO (*Figs 4* and 5) and is overall a safe procedure. In addition, we investigated the relationship between recurrence and tumor size at baseline. In particular, it was found that the more atypical was location, the more likely was the recurrence. This outcome was overall similar to results described in previous studies, implying both RFA [33] and laser ablation [34]. RFA and laser ablation have been shown to be equally effective but with different costs [35]: selection of which treatment for which patient relies on the experience of the interventional radiologist and on the final decision of a multi-disciplinary team. In our experience, RFA grants a rapid pain relief after intervention and a subsequent low impairment of daily life, with a very short hospital stay as well as few post-operative restriction, as well depicted also in a previous paper by Gebauer et al. [25]. This is probably due to the fact that the thermocoagulation of OO using RFA requires only small osseous access to allow insertion of the electrode, thus reducing the loss of bone substance and structural weakening [4].

Among the common complications described in previous papers (skin and muscle burns, infections and nerve lesions) [13], only transient and bearable skin burns and muscle local pain were referred by patients in our series in 5/102 (4.90%) and 1/102 (0.98%), respectively. However, all patients experienced total pain relief and came back to their ordinary daily life within 7 days after RFA. This result was comparable to what previously published by Niazi et al [36].

With regard to imaging guidance, in future navigation systems under CBCT technology will probably help reaching all hard-to-access lesions, thus reducing the impact of the operator experience, sparing time of intervention and dose administered to patients.

About the ablation time, the registered mean total ablation time was 7.2 ± 3.4 min, with a range of 3–15 min. These data were overall similar to previous published papers [35,36].

The limitation of the present study is the single-center retrospective nature. On the other hand, the strengths are the high number of recruited patients, the presence of both typically and atypically located lesions, and the presence of both pediatric and adult patients, thus allowing stratification analysis. Further investigations have already be planned, in particular to better stratify our sample population by age (young and aged patients).

In conclusion, our results provide a sound indication that imaging-guided percutaneous RFA is highly effective in treating OO, voiding the related pain. Its use is advisable due to the excellent cost–effectiveness ratio. Even in patients with atypically located lesions, imaging-guidance grants high rates of technical success, which is the most crucial parameter affecting outcome.

## Supporting information

S1 File (XLS)
